# Effect of ultrasonically-activated irrigation protocols used for regenerative endodontics on removal of dual species biofilm in a three-dimensionally printed tooth model: in vitro study

**DOI:** 10.1186/s12903-024-05415-y

**Published:** 2025-01-18

**Authors:** Saeed Mustafa, Marwa A. Meheissen, Sybel Moussa, Rania ElBackly

**Affiliations:** 1 Endodontics, Conservative Dentistry Department, Faculty of Dentistry, Alexandria University, 13 Champolion St, Azarita, Alexandria Egypt; 2 Medical Microbiology &Immunology Department, Faculty of Medicine, Alexandria University, Alexandria, Egypt; 3 Tissue Engineering Laboratories, Faculty of Dentistry, Alexandria University, Alexandria, Egypt

**Keywords:** Bacterial Reduction, Biofilm, Disinfection, 3D-Printed Tooth Model, *Enterococcus Faecalis*, Passive Ultrasonic Irrigation, Regenerative Endodontics, *Streptococcus Mutans*

## Abstract

**Introduction:**

Eradication of residual biofilm from root canal dentine is critical for the success of regenerative endodontic procedures (REPs).

**The aim of the study:**

To evaluate the influence of ultrasonically activated irrigants in concentrations used for REPs for removal of dual-species biofilm from three-dimensionally printed tooth models with attached dentine samples.

**Methodology:**

Seventy-two three-dimensionally printed teeth models were fabricated with a standardized slot in the apical third of the root to ensure a precise fit with a human root dentine specimen. Dual-species biofilms (comprising *Enterococcus faecalis* and *Streptococcus mutans*) were cultivated in the root canal for a period of three weeks. Models with dentine specimens were randomly assigned into 5 groups according to the irrigation protocol; G1(dis H2O): infected root canals irrigated with distilled water to serve as controls; G2(1.5% NaOCl): 1.5% NaOCl for five minutes; G3(1.5% NaOCl + PUI): 1.5% NaOCl + passive ultrasonic irrigation (PUI) for 30 s; G4(3% NaOCl): 3% NaOCl for five minutes; G5(3% NaOCl + PUI): 3% NaOCl + PUI for 30 s. Bacterial reduction was determined by colony-forming unit (CFU) counting (*n* = 12/G), whilst biofilms were analyzed using field emission scanning electron microscopy in additional samples.

**Results:**

The four experimental groups showed a significant reduction in CFU counts compared to the control group (*p* < 0.05). When compared with (dis H2O), the highest reduction in bacterial count was obtained in G5 (3% NaOCl + PUI) followed by G4 (3% NaOCl), then G3 (1.5% NaOCl + PUI), and finally G2 (1.5% NaOCl).

**Conclusion:**

Results of the current study propose that a 3D-printed mature tooth model can be effectively used to analyze the antimicrobial effects of different irrigation protocols on dual-species biofilm. The use of NaOCl in concentrations used for regenerative endodontics can effectively remove bacterial biofilms. Furthermore, the use of PUI did not significantly enhance antibacterial effects of NaOCl.

**Supplementary Information:**

The online version contains supplementary material available at 10.1186/s12903-024-05415-y.

## Background

Recently, systematic reviews and meta-analyses have demonstrated the promise of regenerative endodontic procedures (REPs) not only for treatment of immature permanent necrotic teeth but for the treatment of mature teeth as well [1, 2].

The permanent loss of pulp tissue during root canal treatment (RCT) comes with several disadvantages, such as loss of sensation, discoloration of the crown, reduced immune defense, altered tooth translucency, and increased vulnerability to root fractures. Furthermore, RCT is a time-consuming and costly procedure for both patients and dentists [3].

REPs of mature teeth have the potential to address these issues by offering a “biological filling” of the root canal with newly generated tissues, restoring both sensory function and immune defense. This approach could potentially reduce the occurrence of flare-ups and may offer greater resistance to fractures compared to traditional methods [3].

The efficacy of REPs is dependent on root canal system disinfection, which is far more challenging to perform in mature teeth than in immature teeth, because the root canal system of mature teeth is more complex, with narrow canals, lateral canals, isthmuses, fins, and ramifications, or several foramina [3].

Residual infection can impede the success of pulp regeneration procedures [3]. Root development has been shown to be hindered by persistence of infection [4]. Residual infection also negatively influences the regeneration of pulp tissue and release of growth factors [5].

Furthermore, persistent apical periodontitis is linked to residual bacteria, commonly in the form of biofilms [3]. Microorganisms are commonly found in multispecies biofilms like *Enterococcus faecalis (E.faecalis)* which can withstand the chemomechanical procedure and endure harsh environments [6]. Recently *Streptococcus mutans* detection in oral sites has been subject to interest, not only because of its primary function in caries initiation, but also because of its link to symptomatically infected root canals [7].

The American Association of Endodontists (AAE) released the ‘Clinical Considerations for a Regenerative Procedure’ [8] which recommends using low concentrations of sodium hypochlorite (1.5%) followed by saline or ethylene diamine tetra acetic acid (EDTA) in the initial visit, and 17% EDTA in the last visit. Sodium hypochlorite (NaOCl) is widely recognized as the most commonly used irrigant in RCTs. Along with its well-established benefits, using NaOCl at low concentrations can effectively eliminate bacteria in infected root canals while minimizing its negative impact on stem cells. However, its main drawbacks include cytotoxicity if it enters peri-radicular tissues and its detrimental effects on stem cell survival and proliferation when used at higher concentrations. It may also accidentally impact the mechanical characteristics and mineral content of dentine [9]. However, the reduced concentration of NaOCl during REPs may interfere with adequate disinfection [10]. Novel techniques have been introduced to enhance disinfection for REPs including nanobubble water that was shown to improve NaOCl penetration within dentinal tubules [11].

Ultrasonically activated irrigation (UAI) with passive tips has also become the most extensively utilized additional approach in root canal disinfection over the last few decades [12]. Indeed, PUI with 1.5% NaOCl has been shown to have no effect on mechanical qualities of human root dentine, according to Elnaggar et al. [9] and has been recommended for safe use in regenerative endodontics [9]. However, the microbiological impacts of combined use of PUI and variable irrigation periods have not been studied in a regenerative endodontic setting.

Furthermore, to assess the elimination of intracanal biofilms, several models have been devised, with most research using single-rooted teeth. Although there is no concrete evidence to support this approach, standardizing the root canals’ dimensions and curvature in human tooth models remains challenging. Thus, the creation of a standardized model has become necessary by integrating advanced computerized technologies to enhance human tooth models. This novel experimental model was used to investigate the effectiveness of different UAI systems for removing multi-species intracanal biofilms using a standardized three-dimensional (3D) printed model with curved canals [13].

The use of a 3D printed mature tooth model with an attached dentine specimen not only offers standardization and simulation of the clinical condition, it additionally incorporates natural dentine as the substrate for the biofilm while standardizing all other variables. Furthermore, it can be a valuable model for testing of regenerative endodontics protocols where emphasis is more on chemical disinfection rather than mechanical instrumentation.

Thus, the rationale for this present study was to evaluate the influence of ultrasonically activated irrigants in concentrations used for REPs for removal of dual-species biofilm from three-dimensionally printed tooth models with attached dentine samples.

The null hypothesis was that there would be no difference between different irrigation protocols on removal of dual-species biofilm.

## Methods

### Study design

**Fig. 1:**
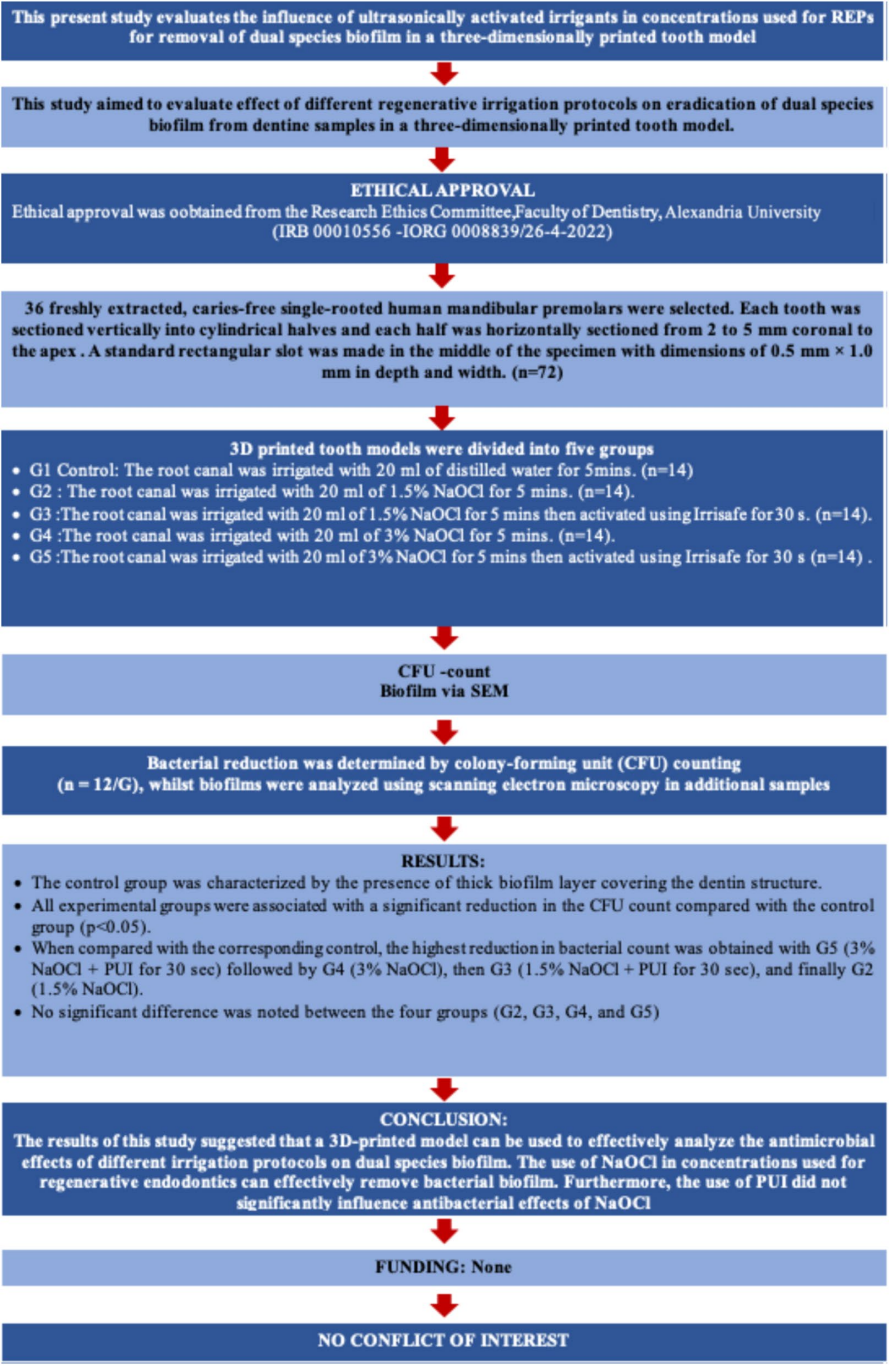
Study design according to PRILE guidelines

The current laboratory in vitro study was conducted and reported in full accordance with the Preferred Reporting Items for Laboratory studies in Endodontology (PRILE) 2021 guidelines [14] (Fig. 1).

**Fig. 2:**
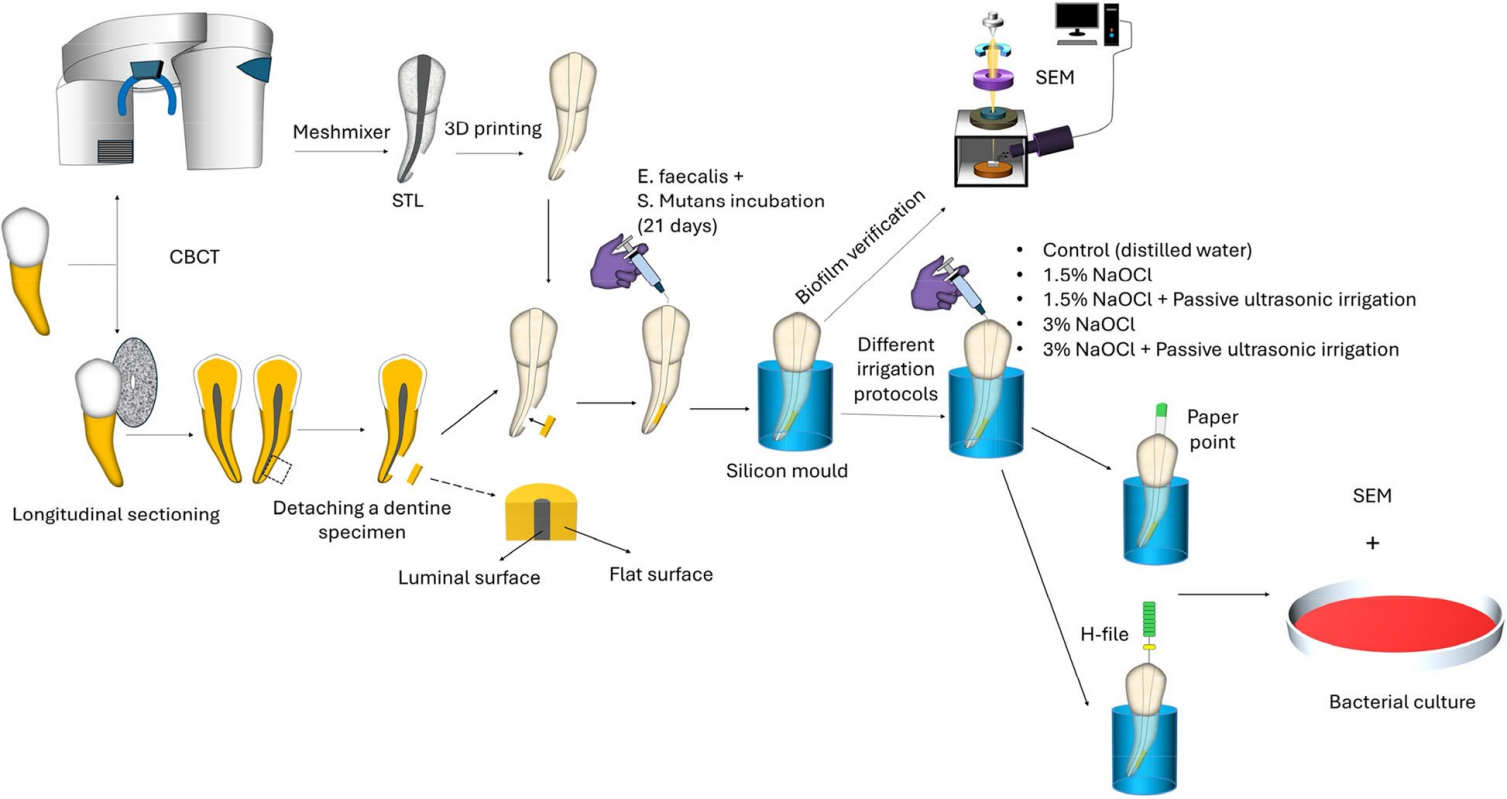
Schematic diagram of methodology executed in the current study

This study was performed on 36 extracted human single-rooted mandibular premolar teeth, which were obtained from the outpatient clinic of the Oral and Maxillofacial Surgery Department due to periodontal or orthodontic reasons, all having fully developed root apices. Ethical approval was granted from The Institutional Ethics Committee of the Faculty of Dentistry, Alexandria University, Egypt (IRB 00010556-IORG 0008839/26–4-2022). Written informed consents were obtained from all participants and/or legal guardians, who agreed to donate their teeth for this study. A detailed description of the methodology executed in the current study is graphically presented in (Fig. 2).

### Design of simulated root canal model [13] (Fig. 3)

**Fig. 3:**
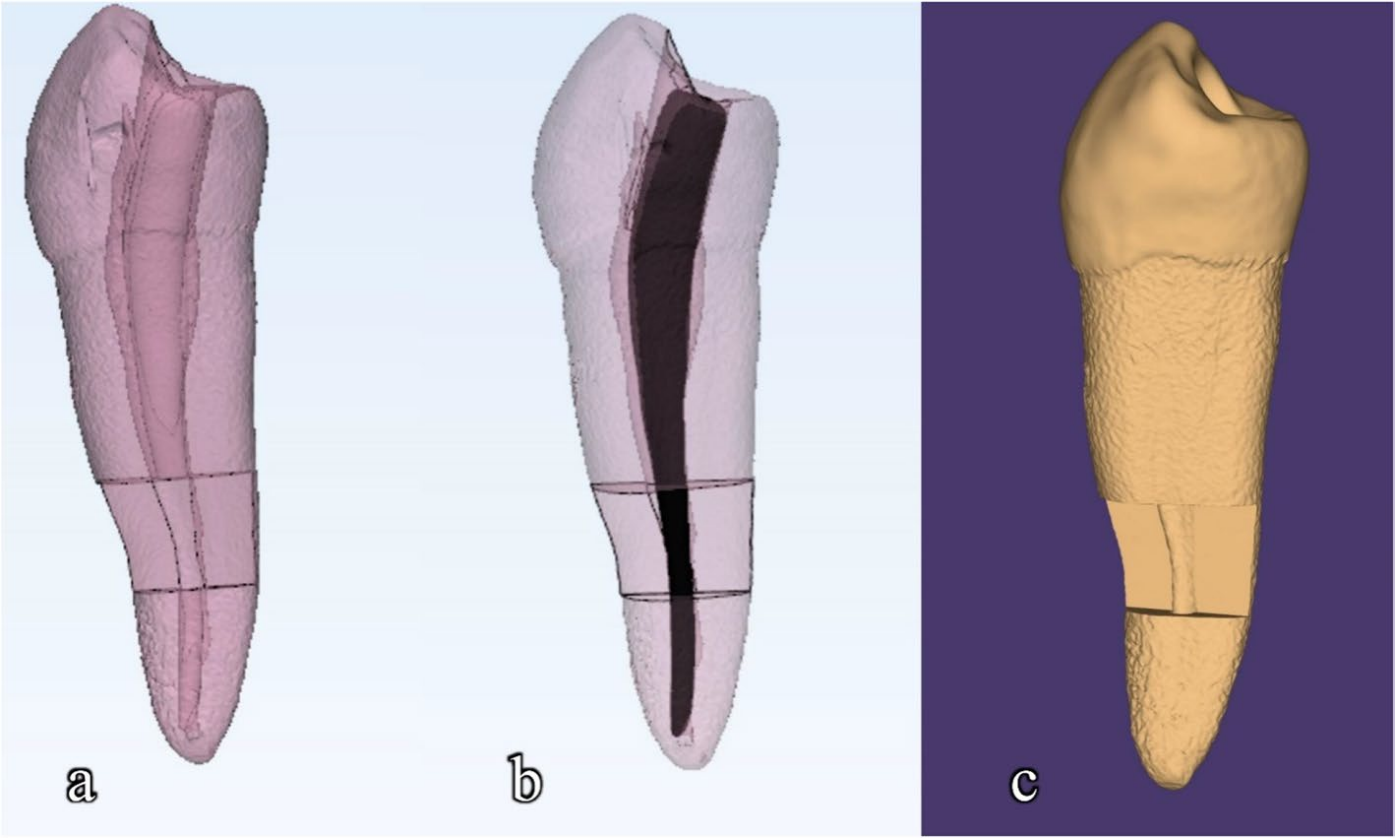
Shows the design of the simulated root canal model (**A**, **B**) 3D editing software (Meshmixer) (**C**) stl file

An intact mandibular second premolar with a 20° curvature was chosen from a pre-existing cone-beam computed tomography (CBCT) examination. A CBCT scan was obtained with PaX-i3D SMART (VATECH, Gyeonggi-do, Korea) which has a resolution of 0.2 mm voxel. The CBCT exposure parameters were set to 80 kVp, 5 mA, and 18 s, with a field of view measuring 120 × 90 mm. The patient’s data were obscured, and this was taken from an anonymous patient from the patient records of the Conservative Dentistry Department. The root canal was designed to feature an apical foramen size of 0.30 mm with a continuous 0.06 taper using a 3D editing software (Meshmixer; Autodesk, San Rafael, CA, USA) [13].

The inner side of the curvature was adjusted 2 to 5 mm coronal to the apex to create space for placing the dentine specimen (coronal side width = 5.4 mm, apical side width = 4.5 mm, height = 3.0 mm, and thickness = 1.1 mm). The 3D model of the sample was exported in STL format to a 3D printer (Sonic Mini 4 K; Phrozen, Hsinchu, Taiwan). A total of 72 3D-printed tooth models were utilized for the study [13].

### Preparation of dentine specimens [13] (Fig. 4)

**Fig. 4:**
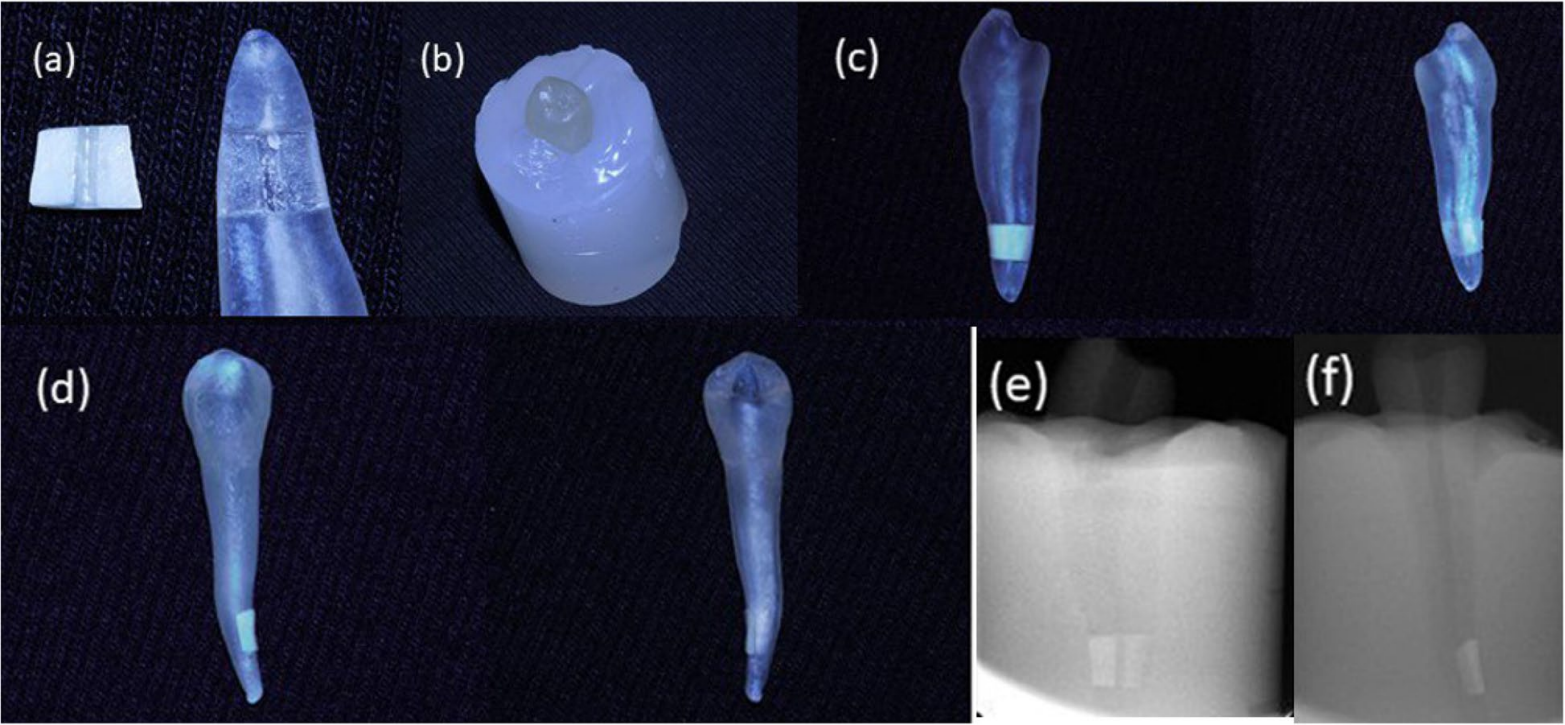
Shows a 3D-printed artificial tooth model with attached dentine specimen (coronal side width: 5.4 mm, apical side width: 4.5 mm, height: 3.0 mm and thickness: 1.1 mm). **a** Dentine specimen with 3D model. **b** The model placed in silicone mold. **c **Proximal view. **d** Labial view. **e** Periapical xray (mesial aspect). **f** Periapical xray (labial aspect)

A total of 36 extracted human single-rooted mandibular premolars were preserved in saline until specimen preparation. Mandibular premolar teeth with single roots, mature apices and single canals with a 20° curvature (Schneider, 1971) [15] were included in the study. Multirooted teeth, teeth with open apices, cracks, calcification, root caries, fractures, external or internal resorption and previously endodontically-treated teeth were excluded.

A diamond saw (Micracut 150, Metkon Metallography, Bursa, Turkey) was used to section each tooth vertically buccolingually into two cylindrical halves, with each half then axially sectioned 2 to 5 mm coronal to the apex for standardization of obtained specimens. To fit the dentine specimen in the 3D-printed tooth models, the pulpal side of each dentine specimen was polished with 800 grit silicon carbide (SiC) paper and refined with Sof-Lex discs (3 M ESPE, St. Paul, MN, USA) (Fig. 4 a) (*n* = 72 dentine specimens).

Then, in the middle of each specimen, a standard rectangular slot with depth and width of 0.5 mm and 1.0 mm, respectively was made to simulate the root canal. An ultrasonic instrument (ET18D tip; Acteon, France) was used to prepare a guiding groove, and a ceramic stone was used to scrub the slot in order to achieve the desired size according to the dimensions of the 3D-printed model.

The dentine samples were inserted into the 3D-printed tooth models using friction fit, without intervening materials, and the outer surface was sealed using a cyanoacrylate-based adhesive after immersion of each specimen in 17% EDTA for one minute to remove the smear layer (Fig. 4 a, c). No material was removed from the canal walls to mimic clinical practice in accordance with regenerative treatment protocol emphasizing further the importance of extensive irrigation and activation procedures*.* To create a closed-end system, the root was embedded in polyvinylsiloxane impression material (Charm Flex; Dentkist, South Korea) (Fig. 4b). All samples were autoclaved for 30 min at 121 °C.

### Intra-canal biofilm formation [16]

*E. faecalis* (ATCC 29212) and *Streptococcus mutans* (ATCC 25175) culture in brain heart infusion broth (BHI) were prepared and adjusted to a concentration of 3 × 10^8^ CFU/ml spectrophotometrically to match the turbidity of one McFarland scale to ensure equal bacterial inoculation of dual bacterial suspension.

**Fig. 5:**
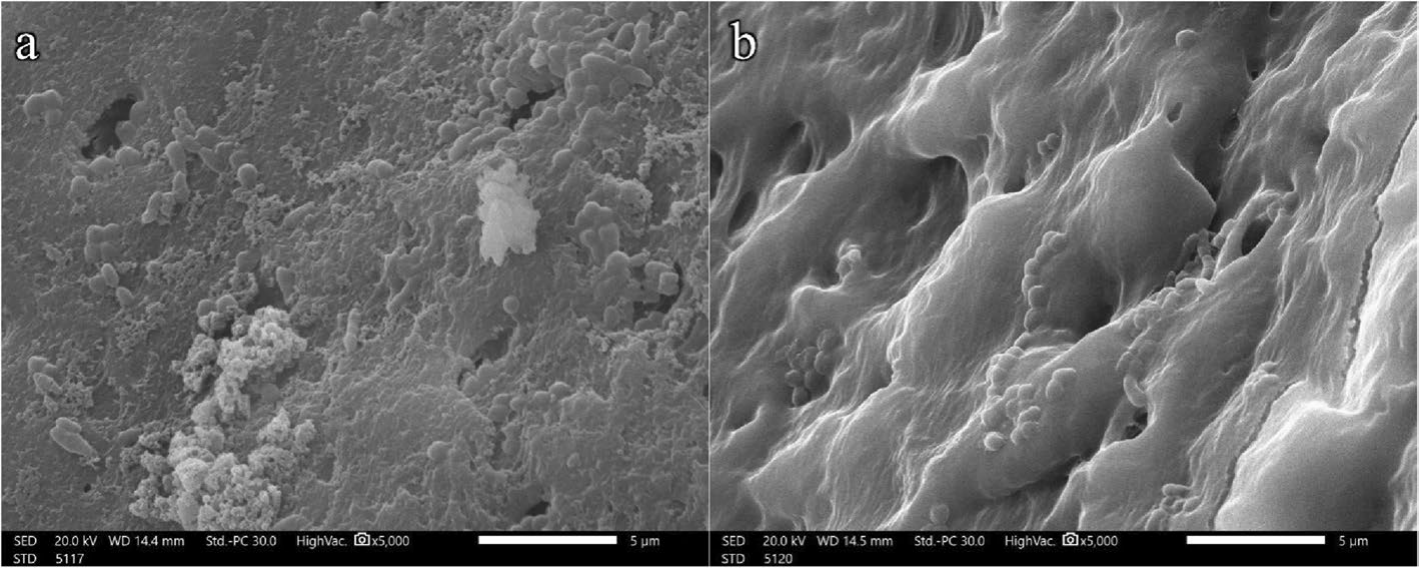
SEM images of 3-week-old dual biofilm showing formation of a mature biofilm encrusting the dentine surface. **a** Luminal surface of dentine specimens (magnification × 5000). **b** Surface of dentine specimens in contact fit with model (magnification × 5000)

The prepared sterilized root canals were injected with mixed equal suspensions (1:1) of *E. faecalis* and *Streptococcus mutans* using a sterile 1 ml insulin syringe until the root canals were filled completely at the level of canal entrance. All samples were inoculated under aseptic aerobic conditions in a class II biosafety cabinet (BIOAIR, Italy) to prevent airborne bacterial contamination. All samples were incubated under aerobic conditions for three weeks at 37 °C. The mixed inoculum inside the canal was replaced every other day by a fresh suspension. At the end of the incubation period, two samples were randomly selected for quantitative culture to exclude microbial contamination and confirm dual biofilm formation with *E.faecalis* and *Streptococcus mutans*. Two random samples were retrieved and examined by SEM to ensure mature biofilm production (Fig. 5).

### Experimental procedures [8, 9, 13]

Delivery of the irrigants was performed using positive apical pressure with a 3-mL luer-lock syringe and a 27-gauge side-vented needle (KDL, China). The needle’s tip was placed 3 mm short of the apex. A flow rate of 1 ml per 10 s was maintained for 30 s, utilizing up-and-down motions without any binding. The investigator prepared 72 envelopes, each containing a concealed group code according to a list of computer-generated random numbers, to be sequentially assigned to the samples. The operator opened each envelope and labeled the corresponding cryovial with its assigned code, ensuring rigorous randomization with an equal allocation ratio [17]. The samples were then divided into five groups as follows:

(i) (dis H2O) group = Irrigation using 20 ml of distilled water for five minutes, and then distilled water was left in the canal for an additional five minutes (*n* = 14).
(ii) (1.5% NaOCl) group = Irrigation using 20 ml of 1.5% NaOCl for five minutes (*n* = 14).
(iii) (1.5% NaOCl + PUI) group = Irrigation using 20 ml of 1.5% NaOCl for five minutes, followed by activation using Irrisafe for 30 s in conjunction with a Satelec P5 Newtron XS ultrasonic unit (Acteon) set to a power level of 6. The tip was positioned 2 mm short of the apex, and up-and-down movements were performed (*n* = 14).
(iv) (3% NaOCl) group = Irrigation using 20 ml of 3% NaOCl for five minutes (*n* = 14).
(v) (3% NaOCl + PUI) group = Irrigation using 20 ml of 3% NaOCl for five minutes, followed by activation using Irrisafe for 30 s (*n* = 14).

For PUI groups, no replenishment of the solution was performed during passive ultrasonic activation.

Finally, all groups were irrigated with saline and the final irrigation was performed with EDTA (20 mL/canal, five minutes), with the irrigating needle placed about 1 mm from the root end in an up-and- down motion without binding. To neutralize the effect of NaOCl, the specimens were treated for 1 min with 1 ml of 4% sodium thiosulfate (Piochem, Egypt).

### Sampling from inside the root canals [16, 18, 19]

Double blinding was implemented, where both the microbiologist and the investigator were not aware of the group allocation. Sampling was done immediately after treatment protocols of all experimental groups and controls. Each root canal was filled with 1 ml of sterile saline solution, and three sterile paper points of size #35 were utilized for each specimen. The paper points were left in the canal for one minute before being transferred to a corresponding sterile Eppendorf tube (Eppendorf-Elkay, Shrewsbury, MA, USA) containing 1 ml of sterile saline.

Additionally, a sterile H-file #35(MANI, INC, Japan) was used to file the dentinal walls vigorously for 20 s. Care was taken to minimize contact between the H-file and the resin canal wall to avoid any confounding results due to inappropriate sampling. The resultant dentine chips were suspended using sterile saline in the same Eppendorf tubes previously used for the paper points to assess bacterial count.

### Bacterial semi-quantitative culture (CFU/ml) [20]

Eppendorf tubes were vortexed for 20 s. Seven serial tenfold dilutions (10^–1^-10^7^) were performed. Subsequently, 10 μl of each dilution was inoculated onto both blood agar and selective Enterococcus bile esculin agar culture plates (Oxoid, UK) and incubated at 37 °C for 48 h. After the incubation period, the number of CFUs/ml was counted using a colony counter (Fisher Scientific, Waltham, MA, USA).

### Scanning electron microscopy [13, 21]

Two dentine specimens from each group were gently detached from their respective 3D-printed models and fixed in 2.5% glutaraldehyde (Piochem, Egypt). The samples were dehydrated in increasing concentrations of ethanol (10–100 percent) for 30 min, dried by a critical point dryer at room temperature, mounted using carbon paste on a copper stub and gold-sputtered in a vacuum evaporator. The samples were examined using a scanning electron microscope (JEOL JSM-IT 200, Tokyo, Japan) to observe the density and morphology of biofilms.

### Data analysis

#### Sample size estimation

Sample size for 3D-printed teeth was based on 5% alpha error and 80% study power. The log mean (SD) of microorganisms using 1.5% NaOCL with PUI was assumed to be 3.84 (0.55), 4.68 (0.17) for 1.5% NaOCL without PUI and 6.47 (1.55) for untreated samples [22]. Log mean difference in microorganisms was calculated to be 2.63 for 1.5% NaOCL with PUI and 1.79 for 1.5% NaOCL.

According to Alshanta et al. [23] the log_10_ median (minute—max) of microorganisms in untreated samples was 7.74 (7.47 – 7.82) and 5.79 (5.32 – 6.68) for samples treated with 3% NaOCL. Based on Wan X et al. [24]method for means estimation, the mean log_10_ (SD) was estimated to be 7.6795 (0.2013) and 5.9238 (0.7821) for the untreated and 3% NaOCL treated samples, respectively. The log mean difference in microorganism for 5.25% NaOCL without PUI activation was estimated to be 3.072, while the log mean difference for 5.25% NaOCL with PUI was estimated to be 4.5136, suggesting effect size of 46% reduction in microorganisms by employing PUI [22]. Based on comparison of independent means using the highest standard deviation (= 1.55) to ensure adequate power, the minimum sample size was calculated to be 11 samples per group, increased to 13 samples to make up for processing errors. Total sample size = number per group × number of groups = 13 × 5 = 65 samples. The Sample size was based on Rosner’s method [25] calculated using G*Power 3.1.9.7 [26].

Data were analyzed using IBM SPSS, version 23 for windows, NY, Armonk, USA. Normality was checked using Shapiro Wilk test and Q-Q plots.

Log_10_ transformation of bacterial count was done to normalize data, however, normality was not approved. Therefore, data were mainly presented using median and range in addition to mean and standard deviation. Percent reduction in bacterial count compared to the control group was calculated according to the following formula: [(Values of all groups – Values of control groups)/ Values of control groups] × 100. All Comparisons were done using Kruskal Wallis test followed by Dunn’s post hoc test with Bonferroni correction for type I error adjustment. All tests were two tailed and the significance level was set at *p* value < 0.05.

## Results

SEM images showed a mature, uniform biofilm structure covering the whole dentine surface of randomly selected samples before using different irrigation protocols (Fig. 5). For the experimental and control groups, SEM images were taken for luminal surfaces as well as the corresponding surface of the tooth (flat surface; Fig. 2) to assess the bacterial invasion extending into the dentinal tubules.

**Fig. 6:**
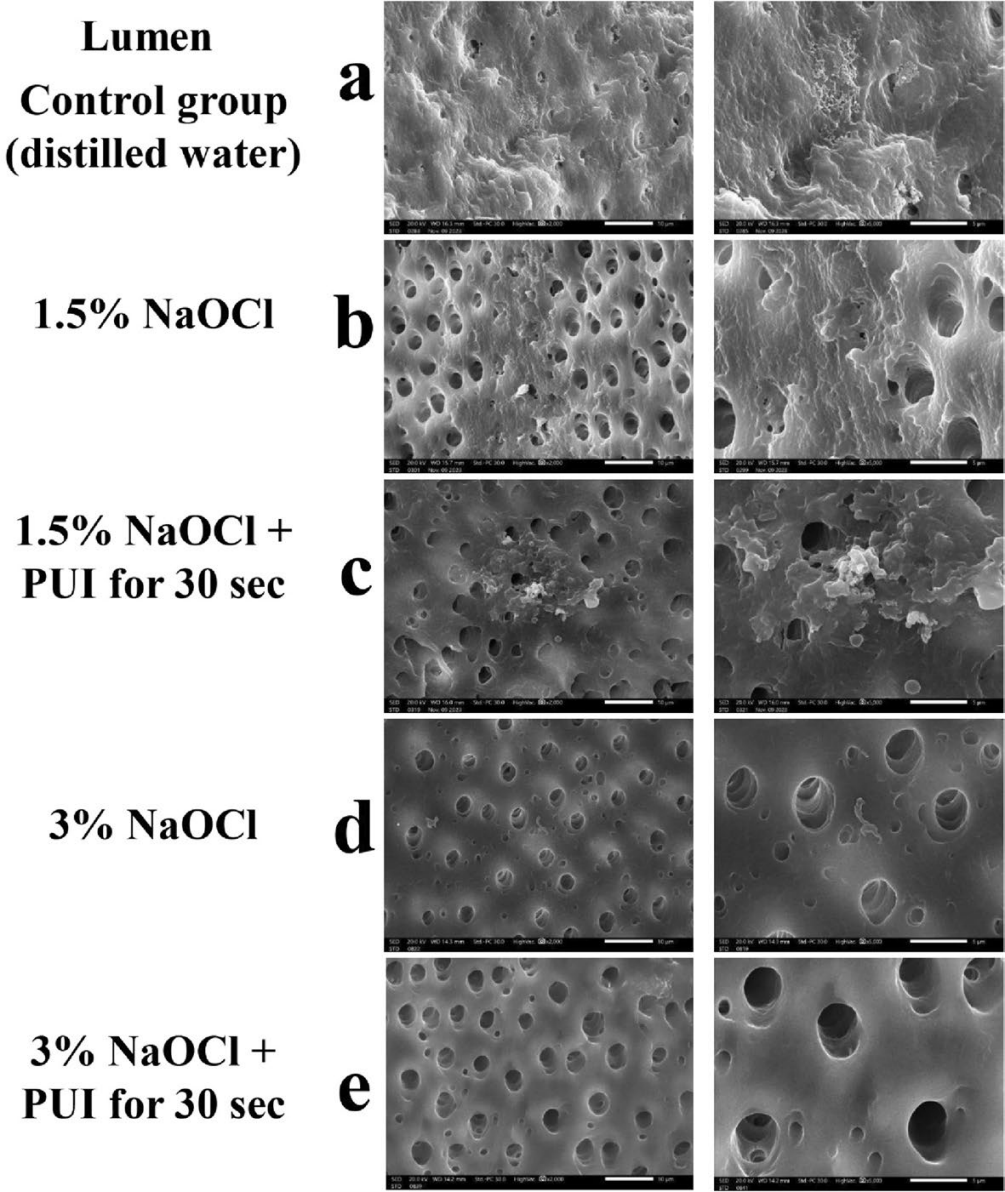
Representative SEM images of 3-week-old biofilms in the luminal surface of dentine specimens treated by different irrigation protocols (× 2000, × 5000). **a** Control, **b** G2, **c** G3, **d** G4, **e** G5. Left panel represents low magnification (× 2000) while right panel represents high magnification for the same groups (× 5000)

**Fig. 7:**
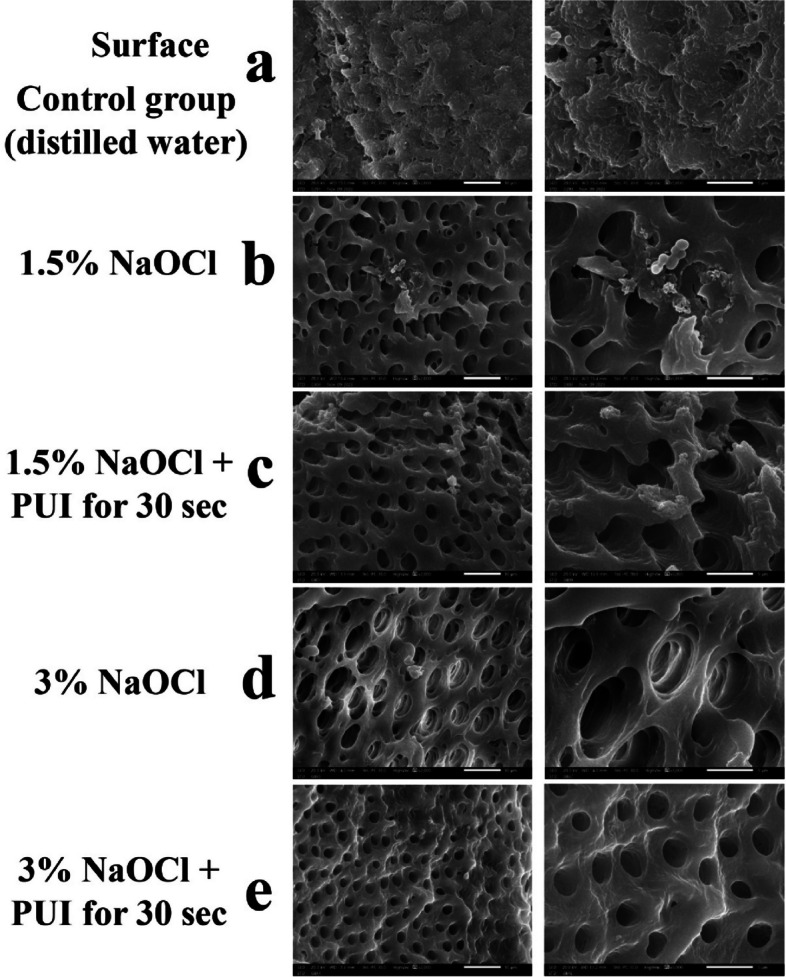
Representative SEM images of 3-week-old biofilms on surface of dentine specimens in contact fit with model treated by different irrigation protocols (× 2000, × 5000). **a** Control, **b** G2, **c** G3, **d** G4, **e** G5. Left panel represents low magnification (× 2000) while right panel represents high magnification for the same groups (× 5000)

SEM images of control specimens showed mature uniform biofilms on both the luminal and flat surfaces. The (dis H2O) group (Figs. 6a, and 7a) showed the highest density and maturity of biofilms almost completely covering the surfaces. Groups (1.5% NaOCl) & (1.5%NaOCl + PUI) showed some residual biofilm (Fig. 6 (b, c), 6(b, c)) however the majority of the surface revealed patent dentinal tubules. As for groups (3% NaOCl) & (3% NaOCl + PUI) (Fig. 6 (d, e), 7(d, e)) these showed the least presence of residual biofilm on the surfaces examined.

**Fig. 8:**
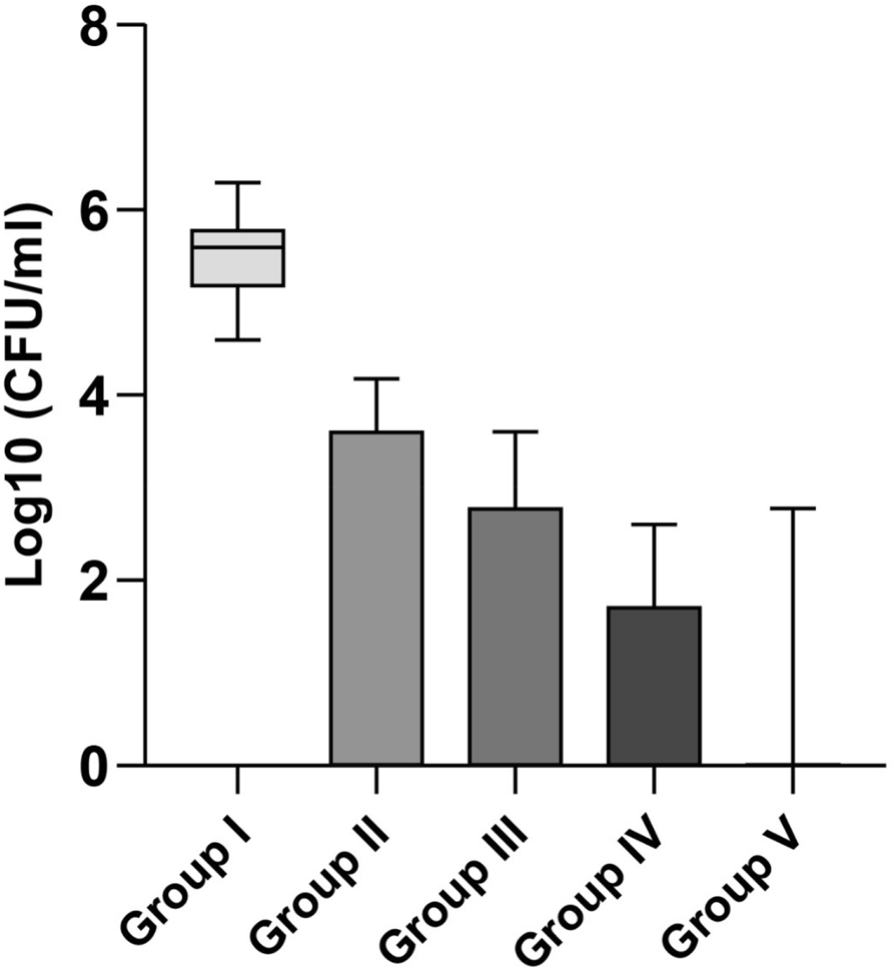
CFU/ml count values for all study groups

Regarding the CFU-counts, the four experimental groups showed a significant reduction compared to the (dis H2O) group (Table 1) *p* < 0.05 (Fig. 8). When compared with the (dis H2O) group, the highest reduction in bacterial count was obtained with (3% NaOCl + PUI) group followed by (3% NaOCl), then (1.5% NaOCl + PUI), and finally (1.5% NaOCl) groups. No significant differences were noted between the latter four groups (Table 2).

**Table 1:** Comparison of bacterial count (CFU/ml) among the study groups

CFU/ml		dis H_2_O (*n* = 11)	1.5% NaOCl (*n* = 11)	1.5% NaOCl + PUI (*n* = 11)	3% NaOCl (*n* = 11)	3% NaOCl + PUI (*n* = 11)
**Count**	Mean ± SD	5.45 ± 5.57 × 10^^5^	2.56 ± 4.62 × 10^^3^	0.74 ± 1.40 × 10^^3^	0.59 ± 1.28 × 10^^2^	0.91 ± 2.07 × 10^^2^
	Median	4.00 × 10^^5^	0.00	0.00	0.00	0.00
	Min – Max	0.040 – 2.00 × 10^^6^	0.00 – 1.50 × 10^^4^	0.00 – 4.00 × 10^^3^	0.00 – 4.00 × 10^^2^	0.00 – 6.00 × 10^^2^
**Log**_**10**_	Mean ± SD	5.51 ± 0.51	1.58 ± 1.86	1.14 ± 1.60	0.6 ± 1.05	0.49 ± 1.09
	Median	5.60^a^	0.00^b^	0.00^b^	0.00^b^	0.00^b^
	Min – Max	4.60 – 6.30	0.00 – 4.18	0.00 – 3.60	0.00 – 2.60	0.00 – 2.78
	H Test (*p* value)	32.77 (< 0.0001^*^)				

^*^Statistically significant difference at *p* value ≤ 0.05, Different superscript lowercase letters denote statistically significant difference

**Table 2:** Pairwise comparison regarding bacterial count (CFU/ml) among the study groups

Groups	Compared to	*p* value
dis H_2_O	1.5% NaOCl	0.002*
	1.5% NaOCl + PUI	< 0.0001*
	3% NaOCl	< 0.0001*
	3% NaOCl + PUI	< 0.0001*
1.5% NaOCl	1.5% NaOCl + PUI	1.00
	3% NaOCl	1.00
	3% NaOCl + PUI	1.00
1.5% NaOCl + PUI	3% NaOCl	1.00
	3% NaOCl + PUI	1.00
3% NaOCl	3% NaOCl + PUI	1.00

^*^Statistically significant difference at *p* value ≤ 0.05

Regarding the effect of PUI, the percent reduction in bacterial count as compared to the control among the study groups, in the (1.5% NaOCl + PUI) group was greater than the (1.5% NaOCl) group and in the (3% NaOCl + PUI) group, it was higher than that of (3% NaOCl) group but these differences were not significant (Table 2).

## Discussion

The current study assessed the effects of using different regenerative irrigation protocols with or without ultrasonic activation on the eradication of dual-species biofilm from three-dimensionally printed tooth models with attached dentine samples.

This research is considered innovative as it employs a standardized 3D-printed model with dentine inserts, which addresses the limitations of earlier studies that lacked comparability due to inherent differences in root canal anatomy, curvature, taper, and apical foramen size, which persist even with standardization in root canal preparation [13].

In this study, human dentine specimens were incorporated into a standardized 3D-printed root canal model with a 20° curvature and a constant dimension (size 30, 06 taper) to provide a real-life substrate [13]. Conversely, the benefits of using human root dentine are absent when employing an artificially fabricated resin root canal model to standardize the root canal geometry [27, 28].

As a result, the model employed in this study offers two benefits: it is standardized and it simulates the clinical environment. This model successfully met the objectives of standardization and clinical setting emulation for the management of intracanal biofilms [13].

Additionally, this model was employed for assessment of disinfection for REPs which are mostly executed in large, oval canals which may add to the challenge of disinfection [29]. Residual biofilms and infected debris can serve as a potential source of persistent infection and treatment failure [29].

In most studies, the root canal biofilm was made up of only one species [30]. This is quite different from the clinical situation where root canal infection is polymicrobial. Compared to mono-species bacteria, multispecies biofilms form thicker biofilms [31] and are more resistant to antimicrobial treatment [30, 32, 33]. Consequently, this work employed a dual-species biofilm typical of the species that are frequently isolated from infected root canals.E. *faecalis* is one of the most frequently identified bacteria in failed RCTs due to its virulence factors [34–36]. This gram-positive facultative anaerobe has strong survival mechanisms [35–37], forming a resilient biofilm that penetrates deeply into dentinal tubules and can survive in harsh conditions, enduring long periods of nutrient scarcity [37, 38]. Similarly, Streptococcus species have been identified as significant contributors to symptomatic apical periodontitis [39, 40]. S. *mutans* has been detected in root canal infections [41] and is known for producing robust biofilms, which enhance its ability to persist and adapt within the root canals [42]. Indeed, the presence of *Streptococcus mutans* has been shown to enhance biofilm formation of *Enterococcus faecalis* [43].

In the current study, counting of bacterial colonies in addition to examination of biofilms was done using SEM. Colony forming unit counts are a commonly used and widely recognized technique for comparing different disinfection methods. The quantity of recovered CFUs has been shown to be highly correlated with both sampling techniques; (paper point and H file) [19]. Indeed, the model used in this study did not interfere with the use of either sampling technique. Therefore, the quantity of planktonic cells extracted from root canals could be a good indicator of the quantity of bacteria that cling to dentine, and vice versa. The sampling techniques discussed have been shown to be sufficient in detection of planktonic and superficial dentine-adherent bacteria that are present in the root canal and on its walls. Additionally, the further use of SEM makes it possible to evaluate the morphology of microbial biofilms and the structure of dentine [19].

All samples were divided into five experimental groups and NaOCl concentrations selected in this study (1.5%, 3%) represented the most frequently used concentrations in regenerative endodontics according to AAE guidelines of irrigation protocols in regenerative endodontics [8]. This recommendation is primarily based on research demonstrating the cytotoxic effects of sodium hypochlorite on the survival of stem cells from the apical papilla in vitro, rather than its ability to eliminate intracanal bacteria in vivo. However, the use of 17% EDTA as a final rinse has been shown to reverse these deleterious effects hence, it was used in the final rinse protocol in the current study [44–47].

PUI was utilized in this investigation to activate NaOCl since it has been demonstrated to be more effective than traditional syringe irrigation at removing pulp tissue and the smear layer [48]. It has also shown superior performance when compared to other dynamic activation systems [49].

PUI was recommended during REPs as a method for complete removal of intracanal medicaments [50] and for efficient activation of EDTA for growth factor release [51]. However, only few clinical regenerative endodontic studies utilized PUI during the disinfection protocol but compared its influence on reduction of microbial species [52].

While the use of PUI may lead to apical extrusion that should be avoided for REPs, our model included simulation of periapical tissues as the root was embedded in polyvinylsiloxane impression material to create a closed-end system and to decrease apical extrusion [49].

To prepare the microorganisms that survived the treatment for microbiological culturing, our goal was to retrieve as many residual bacteria as possible from the canal walls. For this reason, scraping with H-files was done in addition to the use of paper points.

In the current study, use of 1.5% NaOCl (20ml/canal, five minutes) was able to significantly reduce the 3-week-old dual-species bacterial biofilm grown on dentine samples. The percentage of this reduction was different significantly from the control group.

Our results agreed with Swimberghe et al. [19] who reported Significant differences in effectiveness of biofilm eradication among sodium hypochlorite (NaOCl) concentrations of 0.025%, 0.1%, 0.5%, and 2.5% for both anaerobically and aerobically incubated E. *faecalis* biofilms. However, other studies indicate that higher concentrations of NaOCl may be required to completely eradicate bacterial biofilms. For example, Golob et al. [53] suggested that effective and long-lasting decontamination could not be accomplished without using NaOCl at a high concentration of 5%, as bacteria were found to regrow within 48 h following treatment with 1% and 3% NaOCl. Additionally, another study demonstrated that while 1% NaOCl only partially reduced E. *faecalis* viability in the biofilm, concentrations of 2.5% and 5.25% NaOCl completely inhibited bacterial growth [54]. Regarding the effects of 1.5% NaOCl in the current study, Tagelsir et al. [55] found that 5-min biofilm exposure to 1.5% NaOCl provided a significant antibiofilm effect that could eliminate a substantial amount of 3-week-old *E. faecalis* biofilm.

It is noteworthy to mention that a marked difference in eradication of bacteria was found when using 1.5% NaOCl with PUI for 30 s. This demonstrates that PUI can deepen the irrigant penetration into dentinal tubules, enhancing its antibacterial activity, tissue dissolving ability, and smear layer removal [48, 56, 57]. In this study, samples treated with 3% NaOCl (20ml/canal, five minutes) either with PUI or without showed significant reduction and maximum antibacterial effect on a substantial amount of 3-week-old dual biofilm as compared to 1.5% NaOCl.

As for the use of PUI, previous studies compared the effectiveness of ultrasonic activation methods in reducing bacteria compared to conventional needle irrigation [58, 59]. They found that ultrasonic irrigation of 2.5% NaOCl showed statistically significant action in reducing the bacterial load. This is contrary to the findings of the current study where the addition of PUI for 30 s did not have a significant enhancing effect regardless of the NaOCl concentration. This could be due to several factors. It could be due to the unique characteristics of this model since the model was standardized in dimensions thus allowing true estimation of the effect of the variable being tested. This model reproduced a root canal with standard apical foramen size of 0.30 mm and a continuous 0.06 taper for all samples tested. This may exclude the effects of mechanical instrumentation thereby allowing the assessment of irrigation effects only. Hence, needle irrigation in this study may have had accentuated effects providing, alone, remarkable reduction of biofilm after three weeks.

Furthermore, there are multiple variables that may have affected the performance of PUI in this study such as the time of activation. It is also important to note that the time of activation using PUI has been a controversial issue [60]. In the present study, the use of one cycle 30 s activation time was done to simulate clinically relevant protocols [61]. This is contrary to other studies, where ultrasonic activation was done in a larger liquid volume since dentine specimens where used rather than tooth models that simulate natural tooth conditions [19].

Additionally, the effect of vapor lock cannot be denied. It is represented by the generation of air or gas bubbles within a closed-ended system [62]. Although, the ultrasonic tip used allowed deep penetration till the apical third, the dentine specimen fitting within the 3D-printed model may have been subjected to a vapor lock effect in some samples thereby accounting for some variations in the final CFU counts [63].

These variables may have led to lack of significant effects particularly since it has also been speculated that the enhancing effects of PUI may have been overestimated in the literature as they have been largely based on results of in vitro studies and the lack of clinical trials to assess clinical implications [64]. Again, this could be a relevant issue related to the model used in this study which again aimed to more closely simulate clinical conditions thereby providing a more true assessment of the effects of variable tested [19].

However, it remains debatable whether PUI could demonstrate stronger antibacterial activity than conventional needle irrigation [65, 66]. A recent systematic review showed poor evidence regarding the substantially better disinfection capacity of ultrasonic-activated NaOCl irrigants compared to their nonactivated counterparts [67]. Guerreiro-Tanomaru et al. [65] reported that there was no significant difference between conventional irrigation and ultrasonically-activated irrigation with 1% NaOCl.

These results agree with the findings of Bhuva et al. [21], who also showed no significant differences between conventional irrigation with NaOCl and the PUI using this irrigant. Thus, improvement in disinfection was not significant, which corroborates other studies [68]. However, this study confirms previous studies that suggested 1.5–3% NaOCl was adequate as a disinfectant [45].

In the current study it appears that the more influential variable was the concentration of NaOCl rather than the PUI although there were no significant differences between any of the experimental groups. However, the study’s null hypothesis was rejected, as there were significant differences between the control group and different irrigation protocols on removal of dual-species biofilm.

Nevertheless, this study had some limitations. There was some variability in the readings of different samples within the same group which may have resulted in a lack of statistical significance. This could be due to variability of samples obtained from different tooth specimens with different ages despite standardization of the model itself. Another limitation of the current study could be related to sampling from the dentinal specimens alone. Previous studies have shown that methacrylate-based root canal models could be successfully used to grow dual-species biofilms [27]. Hence, we cannot exclude that biofilms were also formed on the resin canal surfaces. However, the objective of the current study was to use this model to standardize other parameters and evaluate solely the effect of the different irrigation techniques on produced biofilms on dentinal surfaces not on resin surfaces particularly since human dentine is the most preferred substrate for bacterial biofilm studies [19]. While this study employed a dual-species biofilm to further simulate the clinical condition more precisely, it is possible that the actual CFU count before and after treatment as well as the thickness and the diversity of the biofilms were influenced by the interaction between the two species. However, the main objective of the study was eradication of total biofilm rather than identify the effects on a single type of bacteria per se.

This study highlighted the clinical relevance of using a 3D printed mature tooth model to better simulate the clinical situation. Furthermore, it highlights that thorough disinfection during root canal treatment is crucial particularly when targeting strategies that can be effective for regenerative endodontic applications.

Future studies can evaluate the reduction of bacteria of multispecies biofilm using the 3D-printed tooth model yet with different activation techniques in conjunction with concentrations used for REPs. Future recommendations should also assess the effect of irrigation techniques on narrow and curved canals and use confocal laser scanning microscopy to quantitatively and non-invasively analyze the antibacterial efficacy of different regenerative irrigation protocols.

## Conclusion

Findings of the current study suggest that a 3D-printed mature tooth model can be used to effectively analyze the antimicrobial effects of different irrigation protocols on dual-species biofilm. The use of NaOCl in concentrations used for regenerative endodontics (1.5–3%) can effectively eradicate bacterial biofilms. Furthermore, the use of PUI did not significantly enhance the antibacterial effects of NaOCl.

## Supplementary Information

Supplementary Material 1.

## Data Availability

The data and materials collected in this study are available from the corresponding author when requested reasonably.

## Abbreviations

REPs: Regenerative endodontic procedures
RCT: Root canal therapy
*E. faecalis*: *Enterococcus faecalis*
CFU: Colony-forming unit
AAE: American Association of Endodontists
UAI: Ultrasonically activated irrigation
PUI: Passive ultrasonic irrigation
PRILE: Preferred reporting items for laboratory studies in endodontology
CBCT: Cone-beam computed tomography
EDTA: Ethylenediaminetetraacetic acid
NaOCl: Sodium hypochlorite
BHI: Brain heart infusion
