# Supplementary material for: Effect of ultrasonically-activated irrigation protocols used for regenerative endodontics on removal of dual species biofilm in a three-dimensionally printed tooth model: in vitro study
Source: BMC Oral Health. 2025 Jan 18;25:98. doi: 10.1186/s12903-024-05415-y (PMC11743002; doi:10.1186/s12903-024-05415-y)
Supplement: Supplementary file 1 — Supplementary Material 1. [file 12903_2024_5415_MOESM1_ESM.docx]

**Supplementary Table 1: Bacterial count (CFU/ml) for the study groups.**

|  | Group I  (distilled water ) | Group II  (1.5% NaOCl) | Group III  (1.5%NaOCl+PUI) | Group IV  (3% NaOCl) | Group V  (3% NaOCl+PUI) |
| --- | --- | --- | --- | --- | --- |
| 1 | 150000 | 4000 | 500 | 400 | 600 |
| 2 | 2000000 | 6000 | 3000 | 200 | 400 |
| 3 | 400000 | 200 | 600 | 50 | 0 |
| 4 | 600000 | 3000 | 4000 | 0 | 0 |
| 5 | 350000 | 15000 | 0 | 0 | 0 |
| 6 | 1000000 | 0 | 0 | 0 | 0 |
| 7 | 500000 | 0 | 0 | 0 | 0 |
| 8 | 600000 | 0 | 0 | 0 | 0 |
| 9 | 300000 | 0 | 0 | 0 | 0 |
| 10 | 40000 | 0 | 0 | 0 | 0 |
| 11 | 50000 | 0 | 0 | 0 | 0 |
